# Report of the Tuberculosis Committee*United States Veterinary Medical Association, October, 1893.

**Published:** 1893-12

**Authors:** A. W. Clement


### REPORT OF THE TUBERCULOSIS COMMITTEE.*

By A. W. Clement, V.S.

Mr. Chairman :—The subject of this communication, tuberculosis, is now universally admitted to be an infectious disease, manifested by definite clinical symptoms and characterized by certain anatomical changes in the tissues, due to a specific micro-organism known as the tubercle bacillus.

The first important study of the disease was made in the first part of the present century, when Boyle and Läennel declared tuberculosis to be a separate affection, due to the deposit of tubercles. a specific product independent of ordinary inflammation.

Then followed the discovery of inoculation by Läennel, the inoculation experiments of Villemin, the diagnosis of pulmonary tuberculosis by the presence of elastic tissue in sputa of phthisical patients, and finally the most brilliant achievement of modern medical science, the discovery by Koch, in 1882, of the cause of tubercle and the etiology of the disease. This brilliant discovery set at rest all doubts as to the nature of the disease and proved beyond all question the indentity of the disease in all animals.

The tubercle bacillus gives rise to anatomical changes either in the form of milliary tubercles or in caseous inflammations. It produces secondary infection either by being carried in the blood current to other parts of the body or by traveling along natural channels. The most common form of primary infection in cattle is in a caseous bronchitis or broncho-pneumonia, or in calves more commonly in the form of tabes mesenterica.

The disease is world wide in its distribution and probably affects all classes of animals. It has received much and is daily receiving more and more the attention of governments as an enemy which probably destroys more lives annually than all of the so-called scourges and wars put together. Many conflicting reports have been published as to the extent of the disease among the cattle of this country, but most of them are based upon too insufficient data to make them of much value. We have no regular inspection of herds nor a complete inspection of meat. It is only possible to glean facts relating to the extent and distribution of the disease incidentally, when cattle are examined with other ends in view. Even if we had an organized inspection of herds, the results of such an inspection would not teach us how much tuberculosis is present among our cattle, because the disease is notoriously hard to discover in the living animal, except when advanced,

* United States Veterinary Medical Association, October, 1893.

and breeders and farmers who have had some experience with it are clever enough to remove animals that show the first suspicious symptoms. It is only possible by an extensive examination with tuberculin or through a thorough and well organized system of meat inspection that reliable statistics can be obtained. Some herds have been reported as showing a very small proportion of animals afflicted, while in others the proportion reported has been frightfully high.

We are warranted in saying that tuberculosis is quite prevalent in some regions, that occasional cases occur almost everywhere, and that the disease seems to be spreading.

The great dangers from tuberculosis in cattle are, first, its transmission through the meat and milk to man, and, second, its transmission from diseased to healthy cattle.

From the standpoint of public health, both of these questions are of vital importance, for the first depends to a great extent upon the latter. The importance of the maintenance of such a system as would keep the public informed of the dangers from animals suffering with disease communicable to man, demands that our profession be represented on City and State boards of health equally with the medical profession.

While it is quite proper that State Governments should protect its citizens as much as possible against diseases transmissible from the lower animals to man, yet any effort on their part must of necessity amount to but little, without the hearty co-operation of the National Government. Some means for obtaining fairly reliable statistics of the number of animals affected should be devised and a much more generous spirit should be manifested toward the scientific investigation of these subjects by the Department of Agriculture. The Bureau of Animal Industry should not be hampered by lack of facilities for carrying on the work, and its employees should be chosen on account of their special fitness for the work and not for purely political reasons. Then and then only will the work which can be so well done by this bureau have its effect, and redound to the credit of the veterinary profession of the United States. Though much has been accomplished by the earnest endeavors of many of those connected with the bureau, yet the public has a right to demand, and we believe soon will demand, that the field of research be more fully covered and that it be conducted on purely professional and scientific principles.

Dr. D. E. Salmon, Chief of the Bureau, has very kindly furnished this committee with the advance sheets of Bulletin No. 3, entitled, "Miscellaneous Investigations Concerning Infectious and Parasitic Diseases of the Domesticated Animals," in which appears an article on "Some Experimental Observations on the Presence of Tubercle Bacilli in the Milk of Tuberculous Cows when the Udder is not Visibly Diseased," by Drs. Theobald Smith and E. C. Schroeder.

The milk from six tuberculous cows was inoculated into guinea pigs with the following results:

From cow No. 155, which presented marked symptoms of the disease, and which at the autopsy showed very advanced lesions, five guinea pigs were inoculated, one of which, inoculated two days before the death of the cow, became infected.

Cow No. 156, which had very extensive tuberia, proved negative in the inoculation of four guinea pigs. Two pigs fed with the milk of these cows failed to contract the disease.

Cow No. 233, which had advanced general tuberculosis with slight infection of the udder glands, gave positive evidence in eight guinea pigs and negative results in two.

Cow No. 234, which had extensive tuberculosis, with udder glands normal, gave negative reaction in nine guinea pigs inoculated

Cow No. 303, which presented moderate tuberculosis but with infection of the lymph glands behind the udder, gave negative results in one guinea pig, the only one inoculated.

Cow No. 314, in which tuberculosis was present in a moderate degree, but in which the udder was not affected, gave negative results in the one guinea pig which was inoculated.

To summarize these, positive results were obtained in guinea pigs by the inoculation of the milk from two cows and negative from the other four cows. One of the cows giving a positive result (No. 303) had tuberculosis of the lymph glands just back of the udder. These experiments were conducted under strict antiseptic precautions and are valuable.

Dr A W. Clement, Chairman.

*Dear Doctor:*—In May and June, 1892, I fed two half-grown pigs on milk from a cow with tuberculosis for a period of seven weeks. The pigs were afterwards kept for nine weeks before being slaughtered. Two other pigs from the same litter and of very nearly the same size were kept in the same stable, but in a different pen, and were fed on grain. The four pigs were slaughtered Sept.

30th, 1892. It was found that the pigs which received the milk, having had a balance ration, had grown rapidly and were large and fat, while the pigs fed on grain (principally corn) had suffered from the absence of proteids in their food, and were small and thin. The autopsies showed the two thin pigs, which received no milk, to be healthy, and the two fat pigs, which were given milk, to have tuberculosis of the post-pharyngeal lymph glands, and in one of them the mesenteric glands were tuberculous as well. The post-pharyngeal glands in each case were of the size of a goose egg, cheesy, and with softened creamy centres. The mesenteric glands contained numerous cheesy nodules of the size of a pea. These lesions were shown to be tuberculous by the detection of the bacillus and by guinea pig inoculations.

The cow that supplied the milk was slaughtered, and was found to have extensive pulmonary tuberculosis, but the other organs, including the udder, were healthy.

The second danger from tuberculosis, that which relates to the contagiousness of the disease among cattle, is the side of the question that appeals more especially to the farmer and veterinarian.

An instance illustrating the spread of tuberculosis may be cited from the writer's experience:

A farmer who had conducted a dairy for four years and had never had any disease among his cattle, so far as he was able to discover, purchased a heifer calf, together with some other cattle, in October, 1892.

The heifer was evidently in poor health at the time of purchase and was therefore removed from the farmer's herd, after it had been a member of it for a few days; and "to give it a better chance," it was turned into a passage way in the stable that ran along the front of the cows' heads. Here the heifer was alone, but was able to come in direct contact with the head of every cow in the herd. There was no manger in the passage way, so the calf was fed from the barrow used to carry the food to the cows.

The heifer's appetite was voracious, but it continued to become thinner and weaker, its coat became dry and staring, it suffered with a racking cough, there was an occasional discharge of creamy matter from the nose, and in six weeks from the time of purchase it died. No autopsy was made. Some six weeks thereafter some of the cows commenced to cough; they gradually grew thin and yielded less milk. Early in March a cow died that had been affected as above for some weeks. Before the last of September nine cows had either died or had been sold while still able to walk away. Some of these cows were opened and examined by a veterinarian and were found to have died of tuberculosis. In September, less than one year after the introduction of the heifer, the herd was tested with tuberculin ; 19 animals reacted and were slaughtered and were found to be tuberculous.

In some instances, as the one quoted, the disease spreads with amazing rapidity through almost the entire herd ; in other cases the progress is gradual and slow, and often a herd has harbored it for years and but a small percentage of the animals become affected.

The study of the predisposing causes of tuberculosis of cattle is an important one, and one that is at present quite incomplete, although a number of undemonstrated theories in explanation of this subject have been advanced.

Among the points that require special study are the influences of the following factors on the predisposition to tuberculosis : Heredity, feeding, stabling, climate, season, breed, productiveness, especially in reference to the dairy cow, and age.

It seems probable that excessive stress has been laid on *some* of the above points, since observations show that continued association with tuberculous animals will lead to infection in a large percentage of cases, notwithstanding the other conditions. But they do, to a certain extent, influence the rapidity of the spread of the disease.

Tuberculosis spreads more rapidly in the Winter, when the cattle are housed, than during the pasturing season Some personal observations indicate that the construction of the stalls has some influence on the dissemination of the disease, since it appears to spread more rapidly in stables so arranged that the cows can bring their heads in contact with their neighbors' and touch noses, than in stables fitted with solid partitions between the cows' heads, or with box stalls.

That tuberculosis of cattle is rarely inherited is the testimony of all practicing veterinarians, and is clearly shown by the report of the examination of nearly one million calves in the Munich slaughter house from 1878 to 1882, of which but five were tuberculous, while the percentage of tuberculosis in the old cattle slaughtered ranged from 2—11 per cent. But that heredity plays an important part in predisposition and immunity cannot be doubted.

In one herd tested with tuberculin, each member of a family of five cows reacted, and all were found, upon post-mortem examination, to be affected.

Some cows can mingle with consumptive associates for years and remain free from tuberculosis, and their progeny are usually endowed with the same natural defense.

In another herd in which 40 per cent of the cows had tuberculosis, one old cow and her three daughters proved to be free from the disease.

It has not been shown that the influence of the sire is as powerful as that of the dam in conferring predisposition to and immunity from tuberculosis.

(Signed), LEONARD PEARSON.

While it is now generally admitted and proven by experiments quoted that the milk of tuberculous cattle is exceedingly dangerous to man, the question of infection from the meat supply is in dispute. Some experimentors have produced positive results by feeding of the expressed muscle juice of tuberculous animals, but it is feared that the experimentors may not have used proper antiseptic precautions in obtaining the material. On the other hand, any one who is in the habit of visiting our slaughter houses will observe that the butchers are not at all particular about the knives used in dressing or the cloths used in cleaning the carcasses, but will persistently use the same knife and the same cloths indiscriminately on cattle which are reeking with tuberculosis and those which are healthy.

It is pretty generally contended at present that when the flesh of a bullock is not visibly affected, and when there is not general advanced tuberculosis, the meat may be eaten with comparative impunity. This question, however, requires much further study, and is far from being settled.

### MEANS OF CONTROLLING AND ERADICATING THE DISEASE.

Under this head, Dr. Pearson further writes as follows:

Meat Inspection.—To protect consumer and determine distribution of disease.

Dairy Inspection.—To protect consumer of milk, and remove tuberculous cows from dairies.

After having determined that tuberculosis is present in a herd, it becomes necessary to separate the healthy from the diseased cattle, and thoroughly disinfect the stable.

In the past, the first of these measures has been a matter of the gravest difficulty, since tuberculosis of cattle is excessively difficult to diagnose, except when advanced, and nearly all the beginning cases escape the most thorough and painstaking physical examination. Various methods of surmounting this obstacle to a perfect division of the herd have been suggested; such as an examination of the bronchial mucus, collected through an incision in the trachea with a sponge on a wire, the inoculation of guinea pigs with the milk, the inoculation of such animals with extirpated lymph glands, etc., but all of these methods are time consuming, difficult and unsatisfactory.

Soon after the publication of Koch's work with tuberculin, this substance was employed on cattle to determine its value as a diagnostic agent. It was found that following the administration of a proper dose, a febrile reaction occurred in tuberculous animals, while no effect was produced in animals free from tuberculosis.

In the first summary of results, collected by Eber in 1891, (Centralblatt für Bacteriologie, 9 Nos. 9 and 10,) it was shown that out of 134 animals (cattle) which reacted to tuberculin and were killed, 85.83 per cent. were tuberculous. Of 113 animals (cattle) which showed no reaction after the injection of tuberculin and were killed, 89.38 per cent. were free from tuberculosis. It will thus be seen that in this summary, published very soon after tuberculin was first used for this purpose, there were 14.18 per cent. of failures in the first instance and 10.62 per cent. in the second. We must remember that these experiments were made by a number of experimentors in different places, some skilled and some bungling, all of the autopsies were not carefully made, and, ABOVE ALL, the question of the proper dose was not then settled, and many of the failures may be attributed to that. The most recent foreign reports are almost uniformly favorable.

In my experience of more than 500 cases, of which about 100 have been slaughtered, I can count but one error in diagnosis; an old cow badly diseased, which did not react after a small dose. All of the other results have been most satisfactory. Every cow that gave a reaction and was killed was shown to have tuberculosis.

By the use of tuberculin then, it is possible to isolate the diseased animals and make sure that those remaining are free from tuberculosis. *If the stable is now disinfected*, and the herd retested after an interval of six months, to find cases that might, by some almost impossible chance, have escaped the first examination, we shall have freed the herd from tuberculosis, and it only remains to exclude diseased additions to keep clear of the scourge.

An important question that presents itself is : What shall be done with the cows that react? Our previous experience and present knowledge allow but one answer to this question, and it is, destroy them. All animals that react, ignoring the very few possible exceptions, are tuberculous, but it should be remembered that some of them suffer to but a very slight degree.

In some of the animals we may, upon making the autopsy, find nothing but a few tuberculous areas of the size of a pea, and perhaps these are situated in the lymph glands. In such a condition, an animal cannot scatter the tubercle bacilli, and it might be objected that slaughter is unnecessary waste, but how are we to know that the tubercles, which we are sure exist in the body, are not in the lungs, the kidneys, the uterus, the testicles, or even the udder? The most careful and exact physical examination could easily fail to elicit their presence at some stages of their growth.

We know that nearly all cases of tuberculosis in cattle tend to advance, and that a slight depression or illness may lead to the rapid development of a more general tuberculous condition, starting from the lesion which we *know* is present.

The sale of an animal known to have tuberculosis, though ever so slightly, cannot be justified, either morally or legally, and to keep such an animal in a herd is to harbor a foe of unknown strength.

The writer has never seen the least bad results follow the injection of a quantity of tuberculin sufficient to answer the purposes of diagnosis. It is well known that in a tuberculous man an excessive dose of tuberculin will tend to aid in the distribution of the tubercle bacilli in the body, and thus cause milliary tuberculosis and hasten death. That an excessive dose may do the same in tuberculous cattle is shown by an experiment, in which 0.6 c.c. was administered to a yearling Jersey heifer, that was known, from the marked symptoms, to have pulmonary tuberculosis. The temperature was 101.6 degrees. Following the administration of the tuberculin, a febrile reaction to 106.2 degrees F. came on in 9 hours. The temperature remained above 104 degrees F. for 6 days, when the heifer died, and acute milliary tuberculosis of the lungs, peritoneum, kidneys, walls of the uterus, and all of the lymph glands connected with the thoracic and abdominal organs, was found. This result was evidently due to the large dose, which was six times the normal for an animal of the size of the subject of the experiment.

The normal dose, instead of doing harm, may produce good effects, and I have several cows under observation that gave decided reactions when first tested, clearly indicating the presence of tubercles, but, after receiving three or four injections of tuberculin, the reactions ceased, the general condition of the animals improved, and at present it is impossible to elicit evidence of tuberculosis, either by the physical examination or by the tuberculin test.

August 11th, 1892, a Jersey cow, 5 years old, received 0.25 c.c. tuberculin beneath the skin, and the temperature rose to 2 degrees F. within 12 hours. The cow was in medium condition, had a cough, and her general appearance was a little below the average of the herd.

December 22d, 1892, she received another light dose and reacted $1\frac{3}{5}$ degrees F. within twelve hours. Her condition now improved, she became fatter and her coat became glossy. She was heavy with calf at the time and that may have had some beneficial effect on her general condition.

February 25th, 1893, she calved, and March 1st died of milk fever. An autopsy was made the same day, and the only evidence of tuberculosis was a calcareous deposit as large as a hickory nut in the mediastinal lymph gland, and a hard, fibrous and dry, cheesy area of the size of a large walnut in the tip of the middle lobe of the left lung.

These lesions showed no sign of acute change, the fibrous walls containing the dry, hard, cheesy areas were one-third of an inch thick and exceedingly firm. The calcareous deposit in the mediastinal gland, mixed with dry, cheesy matter, was also surrounded by a very dense fibrous capsule.

Last year, a yearling Jersey bull presented the following symptoms : Condition medium, coat dry, appetite good, temperature 102 degrees F., respiration accelerated, and each accompanied by a grunting sound to be distinctly heard forty feet from the animal ; head extended. In the inter-maxillary space were two hard lumps as large as a goose egg, and two similar lumps, not sensitive to pressure, were high up on each side of the larynx. After an injection of tuberculin (0.1 c.c.) a marked reaction took place, followed in two days by slight improvement in the general symptoms. In three months the bull received 1.2 c.c. of tuberculin in six doses, and improved constantly until the breathing was natural and the enlarged lymphatic glands were reduced to their normal size. The animal was then killed and examined. The thoracic and abdominal organs were normal ; the larynx mucous membrane was normal ; the lymphatic glands, which were felt from without, each contained dry, cheesy masses of the size of a pea, surrounded by dense fibrous membranes, and one of them contained a slight calcareous deposit.

Twelve cows were treated last Spring, each with nine injections of tuberculin, in all about 5 c.c., during a period of fourteen weeks. All had previously reacted to tuberculin and were known to be tuberculous. Nine of the cows ceased to react after two to five injections had been given, but the other three continued to react after each injection. All were killed, and in the nine cows lesions of the greatest variety were found in nearly all of the organs, but all of them—in the lungs, pleura, liver, kidneys, walls of the rumen and peritoneum—were surrounded by fibrous capsules, markedly thicker than are ordinarily found. Three of the nine had but slight lesions, and they were made up almost entirely of dense fibrous tissue, with minute masses of cheesy material at the centres.

The three cows that continued to react showed generalized tuberculosis of both body cavities and affecting, especially, the serous linings of these cavities.

These experiments seem to indicate that tuberculin has a curative action in *some* cases, especially when the disease is slight. Certainly the symptoms frequently improve after the use of tuberculin, and further experiments will be necessary to determine the exact action at the point of disease and the permanency of the relief.

The subject is important enough and the indications that valuable results will be obtained are strong enough to justify the expenditure of a large sum of money on the work. We are indebted to Mr. Jos. E. Gillingham for the financial assistance without which these experiments would have been impossible.

Upon this subject further is the report of Dr. Peters, as follows :

JAMAICA PLAIN, MASS.,

Aug. 10, 1893.

DR. A. W. CLEMENT, Chairman.

*Dear Doctor:*—I propose, in this brief report to you, to state the manner in which the work of eradicating tuberculosis from cattle in New York State has been systematized. As it is, so far as I know, the first State Board of Health in this country to actually make a beginning at grappling with this subject in earnest, I think that a short account of the practical working of the law may prove of interest, together with a few words as to its benefit to the community, errors in diagnosis and the like.

Granting that the New York State Board of Health acknowledges the work already done in investigating the danger to human life from the use of the milk from tuberculous cattle demonstrates it to be unfit as an article of food, there is no need of arguing the pros and cons any further, or of giving details of experiments which have been printed over and over again, and repeatedly quoted.

The law passed by the Legislature of 1892 works admirably. The veterinary inspector is amply protected in his duties by being empowered to call upon a deputy sheriff, or sheriffs, for protection in case he is interfered with, and having a penalty attached for the punishment of any person who obstructs him in the discharge of his duties. The owners of cattle are also protected in their rights by being allowed a claim against the State for any animals destroyed, which is as it should be. It may be argued that a tuberculous cow is valueless, and therefore the owner is not entitled to compensation. This is a very small argument to make when a State kills cattle for the public good. The animals are killed for the benefit of the people, and the owner should be paid in full for the value which they represent to a non-professional eye before slaughter.

The law also provides for the disinfection of stables, cars, boats and the like, a wise provision, especially for application to infected stables. In the enforcement of such a law a system of procedure must in time elaborate itself, and finally, as the result of the combined judgment of Dr. Cooper Curtice, Inspector of Cattle for the New York State Board of Health, the President and Secretary of the Board, and the writer, suitable rules and regulations were prescribed by the State Board of Health and a method of work was adopted, which need not be gone into detail in this report, but which may be ascertained by application to the Secretary of the Board.

After the cattle are tagged, and while the inspector is waiting for the order to kill to arrive, the owners of the creatures calls in one or more neighbors familiar with the value of cattle, who appraise their value, and then swear to the truth of the appraisal before a Justice of the Peace, or a Notary Public. The owner of the animals then writes to the Clerk of the Court of Claims at Albany for instructions for filing his claim against the State, and when he receives his instructions he forwards his application for remuneration, with the certificate that the cattle were killed, and the sworn appraisal to the Clerk of the Court of Claims, and when the Court sits it awards such a sum for the loss of the farmer's stock as seems to it just and proper.

The Inspector of the State Board of Health has nothing to do with appraising the value of any animals killed. It is his duty to see that diseased stock is destroyed, but it is none of his business what it may be worth.

Near New York City the animals were given to a renderer to compensate him for sending men to take them to his rendering works, slaughtering, and helping to make the autopsies. In more rural districts no difficulty was found in finding a man willing to kill a cow and cut her up for her hide. In the former case the carcasses were converted into fertilizer ; in the latter the meat was rendered unsaleable by sprinkling the cadaver with kerosene oil. (An idea of Dr. R. A. McLean's, I believe.) No attempt was made to convert any of the animals into beef, as after they are tagged they become the property of the State of New York, and it was thought that in work of this kind it would not do for the State to attempt to save any money, even by selling the flesh of actually healthy animals killed on suspicion, as it might give rise to unfavorable comments.

The system adopted works admirably in actual practice, and may well serve as a model for other States to copy from in attempts to secure legislation for the suppression of bovine tuberculosis. Where a State Court of Claims does not exist, other arrangements for recompensing owners for cattle destroyed would have to be made, such as having the Legislature make a special appropriation for cattle slaughtered, or something of that kind. Five thousand dollars does not seem a large sum for beginning such an undertaking as provided for in the bill, but it is wonderful how much can be accomplished with such a sum, as fully 20,000 cattle were examined, to say nothing of 125 or 150 autopsies that had to be made. Another Winter a larger sum will be available to continue the good work so well begun.

But little opposition was met with in carrying on the inspection and tagging, and the services of a deputy sheriff were not required more than three or four times, but the work done so far has been chiefly in Orange and Westchester Counties, where the

United States Bureau of Animal Industry was engaged in stamping out contagious pleuro-pneumonia a few years ago, and the farmers and milkmen in these localities were used to having their cattle examined and slaughtered, whether they liked it or not. In a district where there had never been any inspection of cattle and destruction of the diseased, it is possible that more opposition might be met with.

The work is of great value in many ways. It shows—

First. That in a typical dairy farming community, like Orange County, bovine tuberculosis is not as prevalent as many veterinarians believe, there having been only 35 cows killed out of about 10,000 examined; that is, .035 per cent. were found to be diseased under ordinary means of inspection.

Second. Among hardy grade cattle, with a Holstein, Short Horn or Ayrshire cross, kept under ordinary farm conditions, tuberculosis is not as infectious as it is usually believed to be. In every instance but two, only one or two animals were found to be diseased in herds numbering, as a rule, anywhere from twenty to fifty head. In two instances, more than two were found to be diseased in a herd; in one case three were killed; in another five were slaughtered. In Westchester County, out of about 10,000 examined, in the neighborhood of 85 head were slaughtered, but there were more errors in diagnosis made here than in Orange County; a number of mistakes, six or eight perhaps, having been made. In Orange County, out of the 35 cows killed, only one was not tuberculous, and the diagnosis in her case was not positive, as before she was killed it was suspected that she might be suffering from pneumonia, which proved to be the case.

The value of dairy inspection was better demonstrated in Westchester County, as here several herds were found badly infected with tuberculosis. In one instance it was found necessary to destroy an entire herd, not counting a few that died before final arrangements were made for killing the remainder (eleven head having been killed in December); 52 animals all told were slaughtered on two different occasions, one bull, tuberculous, 39 cows, all more or less tuberculous except one, four two-year-olds and eight yearling heifers, 50 per cent. of them being tuberculous. This herd had formerly consisted of fine Jerseys, but a number of so-called natives had been bought during the past few years to take the place of cows that died. The milk from this farm was being sold in New York City as very fine Jersey milk, at the high price of 12 cents a quart.

In another lot of cattle, consisting of 24 head, the milk being peddled in the neighborhood where they were kept, six cows were killed and found to be tuberculous, a total of 25 per cent. In another local milkman's herd, consisting of 15 animals, five were slaughtered and proved to be tuberculous by post-mortem examination, making 33⅓ per cent. of the number on the farm.

The other animals killed in Westchester County were all scattering cases, only one, two or three from a farm, and a few of these which were killed on suspicion proved to be errors in diagnosis. Possible errors of diagnosis were found to be chronic bronchitis, and cases convalescing from catarrhal pneumonia, or capillary bronchitis. Bronchial troubles among cattle were enzootic in Westchester County during the latter part of the Winter and early Spring of the present year, which made the examination of the animals extremely difficult. In many instances it was necessary to re-inspect suspicious cases once, or even twice, at intervals of two or three weeks, before it could be decided whether the trouble was bronchitis or tuberculosis. One animal killed as suspicious, and found to be suffering from chronic bronchitis in one lung, was not destroyed until two months after she was first inspected, yet the piping sound in the lung was as constant at the end of that time as on the first day she was examined.

One case killed on suspicion of being phthisical was found to have a small bit of a brier bush of some kind (apparently either from a raspberry or blackberry bush) in the upper posterior part of the right lung, near the outer border. This bit of stick was between one inch and two inches long, and had been taken into the bronchi with the points of two or three thorns on it directed toward the cow's head, acting as barbs, and preventing it from working in any direction except farther and farther into the lung. It had evidently been in the location where found for some time, as it was surrounded with a calcareous deposit. At the time it was first carried down the trachea, and for some time afterward, it must have caused a great deal of irritation along the course it took.

Another cow was killed as a "roarer," but upon post-mortem examination no tuberculous lymphatic glands could be found around the larynx, pharynx, or in the media-stinum. The trachea was then removed, and it was found to have several of its rings fractured at a point half way between the larynx and thorax, making it oval instead of round, and causing a roaring in respiration which, after any exertion on her part, could be heard for about half a mile across the fields. It is very possible that this accident to the trachea may have been caused in an attempt at some time to smash an apple, turnip, or some similar body in the œsophagus, between a block of wood and a mallet.

Tuberculin as a diagnostic agent was not extensively used, two herds only having been tested with it, one in Westchester County in March, on the herd where all the animals were killed; the other near Poughkeepsie, Dutchess County, in May, the animals being killed early in June. In both herds the results were quite satisfactory, but the latter one was much the better to use for the experiment, as it contained a few healthy creatures, and their general condition was better than in the first instance when it was tried. The Poughkeepsie herd consisted of two bulls and thirty-one cows and heifers, which were tested with tuberculin, besides which were three yearling heifers which were not incoulated, and two young calves. Of the thirty-three animals, all but four cows reacted to the tuberculin, and one was unsatisfactory, as she was not well and had a temperature of 106 degrees at the time of the inoculation. Hence twenty-eight animals out of thirty-three may be looked upon as tuberculous according to tuberculin. A physical examination of this herd indicates one cow with general tuberculosis, one cow and two heifers (one of them a yearling not tested with tuberculin) "roarers" from enlarged tuberculous glands back of the pharynx, and three cows suspicious, because of their coughing and having a bad history connected with the farm.

All the animals that reacted to the tuberculin were killed (and also the heifer that was a roarer, but not tested), and found, in every instance, to be more or less tuberculous. One cow that did not react was killed and found to be perfectly healthy.

The question in this trial seems to be: Is tuberculin too fine a diagnostic agent for use in the examination of dairy herds, unless used as an assistance to our other powers of observation?

One cow had only three small nodules in the liver, another a small cheesy posterior-pharyngeal gland, two or three others tuberculous posterior mediastinal glands. Now the question is; Are such animals any danger to other cattle or to the public health?

No, not in that condition. But, on the other hand, no one can tell when the disease may not become more active, and the inspector may leave behind him an animal as harmless to-day which a few months hence may be in a condition to be a greater danger to the well being of the community.

The tuberculin used in the Poughkeepsie test came from the Bureau of Animal Industry Laboratory, at Washington. I was not present when the tests were made in May, but secured the orders to kill from the Secretary of the New York State Board of Health, and took charge of the slaughtering and autopsies about three weeks later, in June, on which occasion I was assisted by Dr. John Faust, his sons, Dr. Otto Faust, and William Faust, a medical student, and Dr. Norgaard, of the United States Bureau of Animal Industry. These four gentlemen conducted the tuberculin tests about the middle of May.

Although I have always been a skeptic regarding the utility of tuberculin, the experience I have recently had with it has changed my views a great deal, and I now believe that it may be a very valuable agent if properly used.

In suspicious cases, where doubt is felt as to whether an animal is tuberculous or not, it is well worthy of a trial; and in herds where a number of cases of tuberculosis are found, I believe it to be advisable to test the entire herd with it. I do not, however, believe it necessary or practicable to go to every farm in the country and test every single cow to be found with tuberculin, but if a herd be inspected in the ordinary way and found to be healthy, I consider that sufficient.

In a breeding herd no doubt it might be advisable to kill every animal that reacted to tuberculin, no matter what its apparent condition of health might be.

I am yours, very sincerely,

AUSTIN PETERS, M.R.C.V.S.,

*Chief Inspector of Cattle for the New York State Board of Health.*

To summarize this report, your committee concludes that tuberculosis is a world-wide disease, affecting, in all probability, all classes of animals—due to a specific micro-organism known as the tubercle bacillus. That the disease causes great losses in our domesticated animals, and millions of deaths annually in the human family—that the disease may be transmitted from the lower animals to man, through the milk, and under certain circumstances, through the flesh used as food—that the diagnosis is now made possible in all cases by the use of tuberculin.

That the different City and State Boards of Health should have a veterinarian as a member of the board, and that these officers should be in direct and constant correspondence with the proper officials of the Central Government, who in their turn should have final jurisdiction.

That while it may not be practicable, at present, to undertake the complete eradication of this disease, each State should, at least, institute a thorough examination of the herds to determine its prevalence; the question of a radical attempt to stamp it out should be agitated; owners of cattle should be apprised of its dangers, all tuberculous cows should be removed from dairies, and all meat should be subjected to a veterinary examination at the time of slaughter.

